# Clinical study on the optimal rotational stimulation for the unilateral centrifugation subjective visual vertical test

**DOI:** 10.3389/fnins.2025.1529572

**Published:** 2025-02-17

**Authors:** Xueqing Zhang, Qiaomei Deng, Xiaobang Huang, Chao Wen, Wei Wang, Taisheng Chen

**Affiliations:** ^1^Department of Otorhinolaryngology Head and Neck Surgery, Tianjin First Central Hospital, Tianjin, China; ^2^Institute of Otolaryngology of Tianjin, Tianjin, China; ^3^Key Laboratory of Auditory Speech and Balance Medicine, Tianjin, China; ^4^Key Medical Discipline of Tianjin (Otolaryngology), Tianjin, China; ^5^Quality Control Centre of Otolaryngology, Tianjin, China

**Keywords:** unilateral centrifugation, SVV, otolith, utricle, rotational test

## Abstract

**Objective:**

To investigate the optimal stimulation intensity and reference range of the unilateral centrifugation subjective visual vertical (UC-SVV) test and to fill the technical gap in otolithic function evaluation in patients with peripheral vestibular diseases.

**Method:**

Forty healthy subjects (median age 28 years) underwent a UC-SVV test at rotation speeds of 60, 120, 180, and 240 deg./s using the Neuro Kinetics Inc. (NKI) I-Portal 6.0 NOTC rotating chair system. The deviation angles of the UC-SVV line in the centre and left/right positions at each rotation speed were recorded and analysed.

**Results:**

Forty healthy subjects completed the test. The deviation angle of the UC-SVV line differed according to the rotation speed (60, 120, 180, and 240 deg./s). At each rotation speed, the position of the rotation axis (left/centre/right position) affected the deviation angle and direction of the UC-SVV line. At each position of the rotation axis, the rotation speed also affected the deviation angle. When the rotation chair was translated to the left and right positions, the UC-SVV line deviated to the right and left sides, respectively, and the deviation angle increased with increasing rotation speed. The deviation angle at 60 deg./s was significantly less than that at 180 and 240 deg./s (*p* < 0.001). When the rotation axis was in the centre position, the absolute value of the SVV deviation was less than 0.3, and there was no statistically significant difference at any rotation speed.

**Conclusion:**

This study preliminarily discussed the deviation direction and angle of the UC-SVV line under different stimulation intensities in a range of healthy middle-aged subjects. The optimal stimulation intensity for the UC-SVV test is suggested to be 180 or 240 deg./s. Specifically, the peak rotation speed could be 180 deg./s if the subject is unable to tolerate high-speed rotation. This study provides support for improving this technology for evaluation of otolith function and its diagnostic significance in peripheral vestibular diseases.

## Introduction

The vestibular otolith system plays an important role in maintaining spatial orientation and postural stability (Ramos de Miguel et al., 2020). Its pathological changes are not only a common cause of vertigo and balance disorders but also an important indicator of clinical vestibular function (Smith et al., 2024). At present, subjective visual vertical/horizontal (SVV/SVH) tests (Taylor and Welgampola, 2019) and vestibular myogenic evoked potentials (VEMPs) are the main evaluation techniques for the otolithic system in clinical practice (Kantner and Gurkov, 2012; Mueller et al., 2020). In the traditional SVV test, the patient adjusts a vertical line in a dark environment without surrounding reference objects at rest, and the result reflects the bilateral utricle function under static gravitational acceleration (Bragg et al., 2023). Ocular counterroll (OCR) response to head-tilt were recorded and analysed by means of three-dimensional video-oculography in a human rotator to objectively examine the unilateral otolith function (Clarke et al., 1996). While OCR has high requirements for the acquisition of eye movement, and the nystagmus data is easily disturbed. OCR induced by lateroflexion (Kingma, 1997) is more manoeuverable as it does not require large equipment for the evaluation of otolithic function. However, OCR has higher quality control requirements for subjects receiving homogenised stimulation in clinical practice. Helling et al. (2006) indicated that compared SVV in rest and on rotation axis at 240 deg./s indicates then a functional asymmetry of the otolith organs. In their method, rotation stimulated both otolith at the same time, could not evaluate monopleural otoliths function.

The unilateral centrifugal subjective visual vertical (UC-SVV) line test based on rotation is a new detection technology that can apply new stimuli (gravity and centrifugal force) to one utricle on the basis of static gravitational acceleration and dynamically evaluate unilateral utricle function (Gonzalez et al., 2014; Chen et al., 2018). However, the UC-SVV test involves a high rotation speed, which often induces vertigo, nausea and other discomfort in the subject, and is time-consuming. Selecting the optimal rotational stimulation to meet detection needs has become the key to UC-SVV technology. This study evaluated the deviation angle of the UC-SVV line at different rotation speeds in healthy subjects, explored the deviation characteristics of the UC-SVV line at different rotation speeds, and determined the optimal rotation speed to aid in the dynamic evaluation of otolithic function in clinical practice.

## Materials and methods

### Subjects

Forty healthy volunteers, including 17 males and 23 females aged 21–57 years, with a median age of 28 years, were included in this study. All the subjects had no history of dizziness, vertigo, balance disorders, hearing loss, motion sickness, migraine, sleep disorders, or other neurological or mental diseases. There were no activities with adverse effects, such as smoking or drinking. All the subjects had no medication history before the test. All the subjects provided informed consent before their inclusion in the study. The study procedures were approved by the Ethics Committee of Tianjin First Central Hospital.

### Methods

All testing was performed at the Centre for Vestibular and Balance at Tianjin First Central Hospital. The rotational chair device used for the study was a Neuro Kinetics Inc. (Pittsburgh, PA) I-Portal® Neuro-Otologic Test Centre (NOTC) located within a lightproof enclosure (model no. NOTC-O 890819). The participants were guided to sit in the rotational chair and secured with four-point lap and shoulder belts. The participants’ heads were positioned with a moulded foam pad, which included the occiput and cervical neck regions, and a self-centring set of padded arms. All testing was conducted using version 8.0.2 of the VEST™ software of the rotational system.

**Figure 1:**
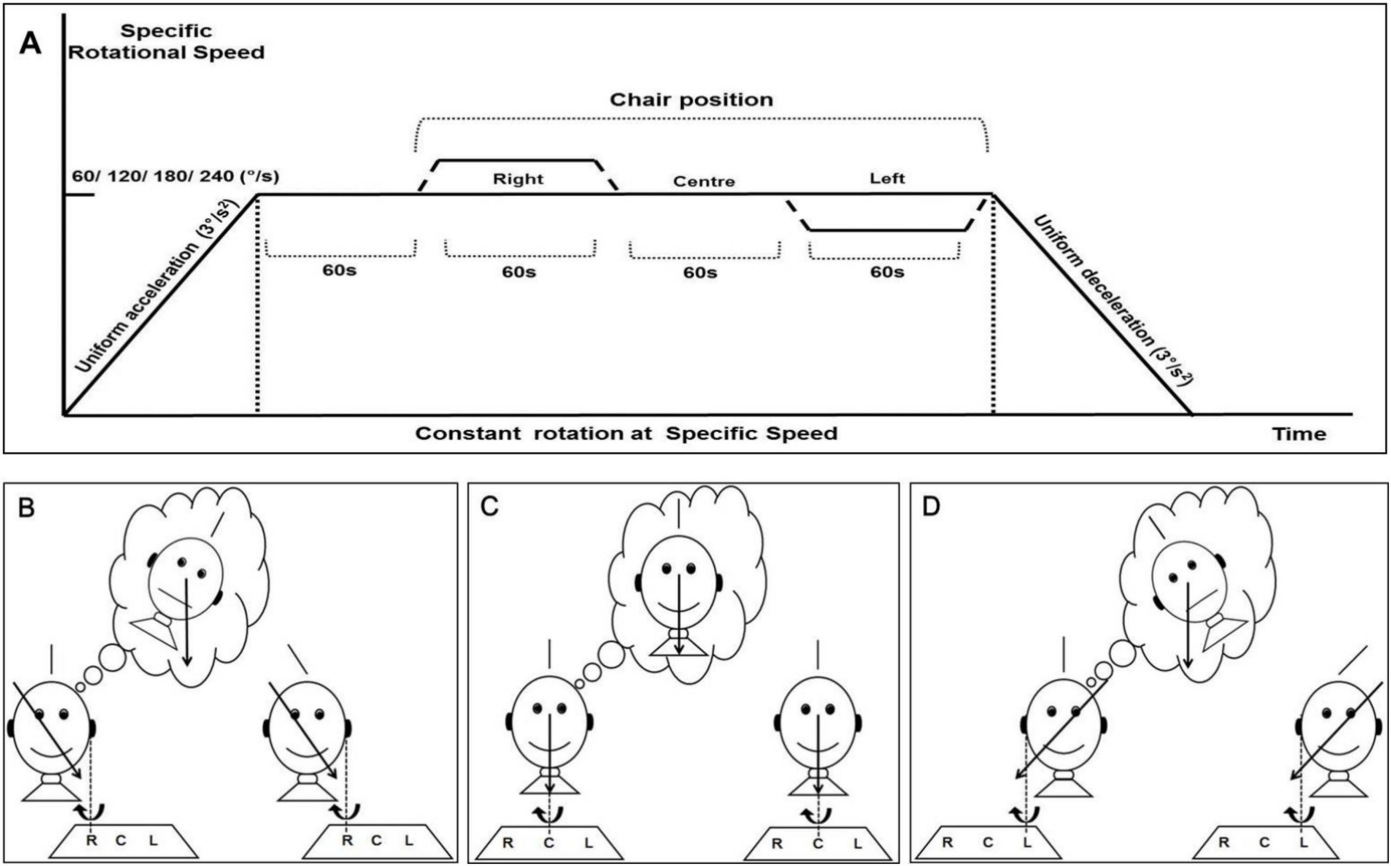
The paradigm of eccentric centrifugation and schematic representation of the oculogravic illusion. **(A)** The chair experienced uniform acceleration, constant rotation, centrifugal rotation and uniform deceleration process. **(B)** When the rotation axis is located at the right position, the “bubble” shows that the subject mistakenly perceives the gravito-inertial acceleration (GIA) direction to be perpendicular to the ground and adjusts the vertical line to tilt to the left. **(C)** When the rotation axis is located at the centre position, the GIA direction is consistent with the direction of gravity, and the “bubble” indicates that the subject accurately perceives that the line is vertical. **(D)** When the rotation axis is located at the left position, similar to the right position, the subject adjusts the vertical line to tilt to the right.

The rotating chair system started in its original position (centre position) and was accelerated clockwise at an acceleration of 3 deg./s^2^ until it reached a specific speed (60 deg./s, 120 deg./s, 180 deg./s and 240 deg./s). After rotating at a constant speed for 60 s, the chair was slowly translated 3.85 cm to the right position while rotating. After continuous rotation at a constant speed for 60 s in the right position, the chair was returned to the centre position. There, the rotation continued at a constant speed for 60 s, and then the chair was slowly translated 3.85 cm to the left position while rotating. After continuous rotation at a constant speed for 60 s in the left position, the chair was returned to the centre position. Each translation time was 30 s. A red laser line randomly appearing 1 m in front of the subject was adjusted to the vertical position at least 5 times during the 60 s of constant rotation in the right, centre and left positions (Figure 1). After each round of testing, the subjects rested for 5 min and then performed another UC-SVV test at another stimulus intensity, using the same method as above. The skewness of the UC-SVV line at each position under different stimulation intensities (60 deg./s, 120 deg./s, 180 deg./s and 240 deg./s) was recorded, and parameters such as the mean and standard deviation were calculated.

### Analysis

Kolmogorov–smirnov test showed that most of the data in this study were approximately normal distribution, so parametric test method was selected for analysis. One-way analysis of variance (ANOVA) was employed to compare differences in direction and deviation angle of the UC-SVV line in the centre and left/right positions at each rotation speed. The data are homogeneity of variance, so Bonferroni correction was used for *post-hoc* analyses. IBM SPSS Statistics 25.0 (IBM, Inc.) was used for the statistical analysis, and *p* < 0.05 was considered a statistical significance. The quantitative data are presented as the means ± SDs and were plotted using GraphPad Prism version 5 (GraphPad, San Diego, California, United States).

## Results

### Angle of UC-SVV deviation in healthy subjects at different rotation speeds

**Figure 2:**
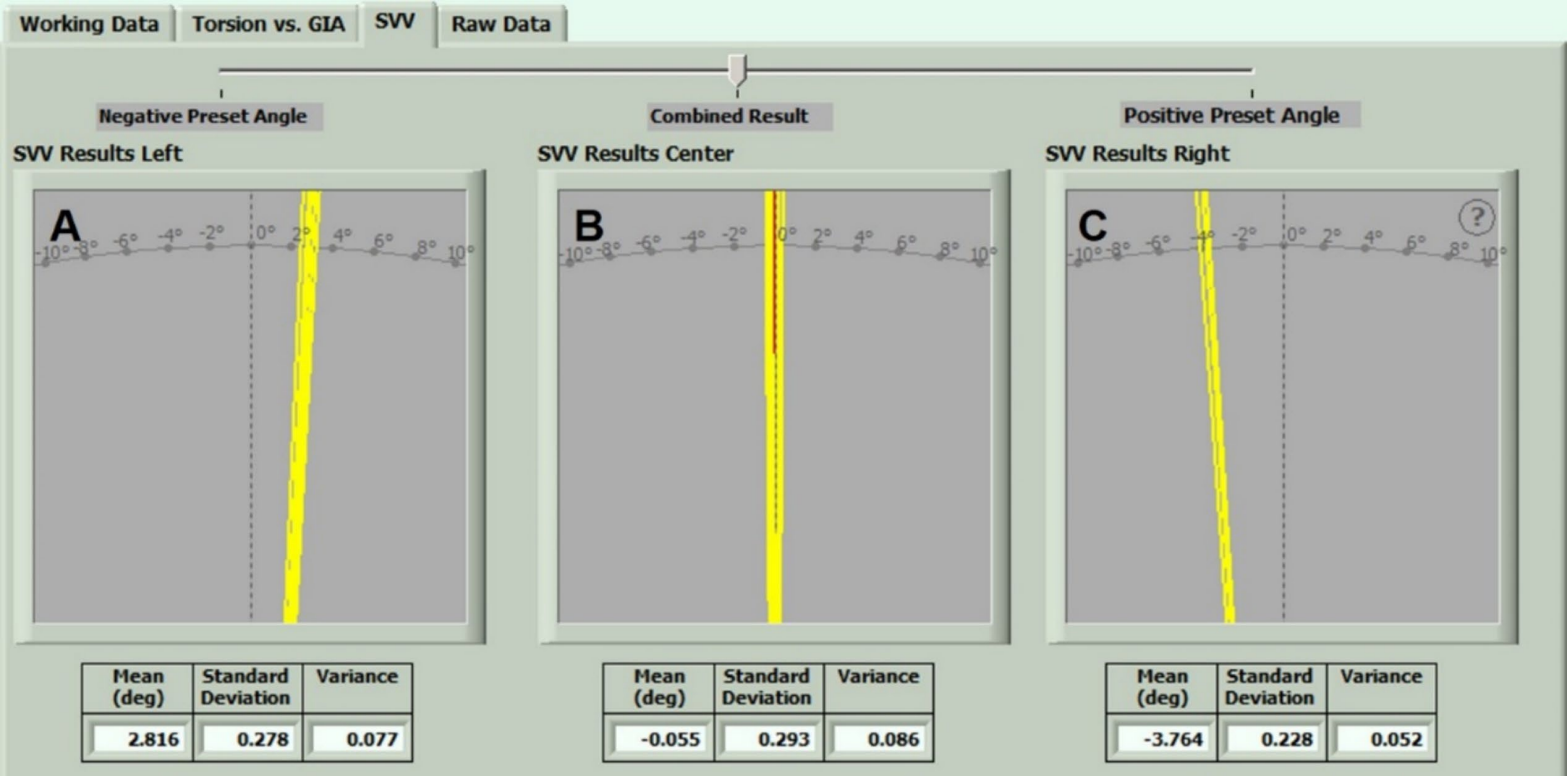
UC-SVV test interface at a peak rotational speed of 240 deg./s. **(A–C)** When the rotation speed of the rotating chair was 240 deg./s, the deviation angle of the UC-SVV line at Left, Centre and Right position, respectively.

The data provided by 40 healthy subjects were considered. The deviation angles of the UC-SVV line at different rotation speeds (60, 120, 180 and 240 deg./s) exhibited a normal distribution. The 95% confidence interval (CI) was calculated, and the details of each parameter are shown in Table 1. The UC-SVV test examples are shown in Figure 2. There was no significant difference between the sexes (*p* > 0.05).

**Table 1:** Parameters of the deviation angles of the UC-SVV line at different rotation speeds in the experimental subjects.

Position	Rotation speed (deg/s)	Mean ± SD (°)	95% CI	Minimum	Maximum	Median
Left Position	60	0.144 ± 1.528	(−0.348, 0.636)	−3.683	3.069	0.175
	120	0.661 ± 2.436	(−0.118, 1.440)	−7.819	4.733	0.996
	180	1.523 ± 2.000	(0.884, 2.163)	−3.130	4.557	1.615
	240	2.341 ± 2.462	(1.553, 3.128)	−3.495	7.949	2.403
Centre Position	60	−0.033 ± 1.578	(−0.538, 1.578)	−4.191	2.527	−0.068
	120	−0.013 ± 2.033	(−0.637, 0.663)	−4.987	3.346	0.146
	180	−0.281 ± 2.621	(−1.119, 0.557)	−11.272	4.750	0.409
	240	−0.253 ± 2.604	(−1.086, 0.580)	−7.110	4.337	−0.097
Right Position	60	−0.185 ± 1.677	(−0.721, 0.352)	−4.991	2.638	−0.048
	120	−0.985 ± 1.947	(−1.608, −0.363)	−5.535	2.477	−0.687
	180	−2.318 ± 2.141	(−3.003, −1.633)	−8.147	2.047	−2.080
	240	−2.976 ± 3.286	(−4.027, −1.925)	−12.368	3.716	−3.022

### Influence of different rotation positions on the deviation angle of the UC-SVV line at a specific rotation speed

**Figure 3:**
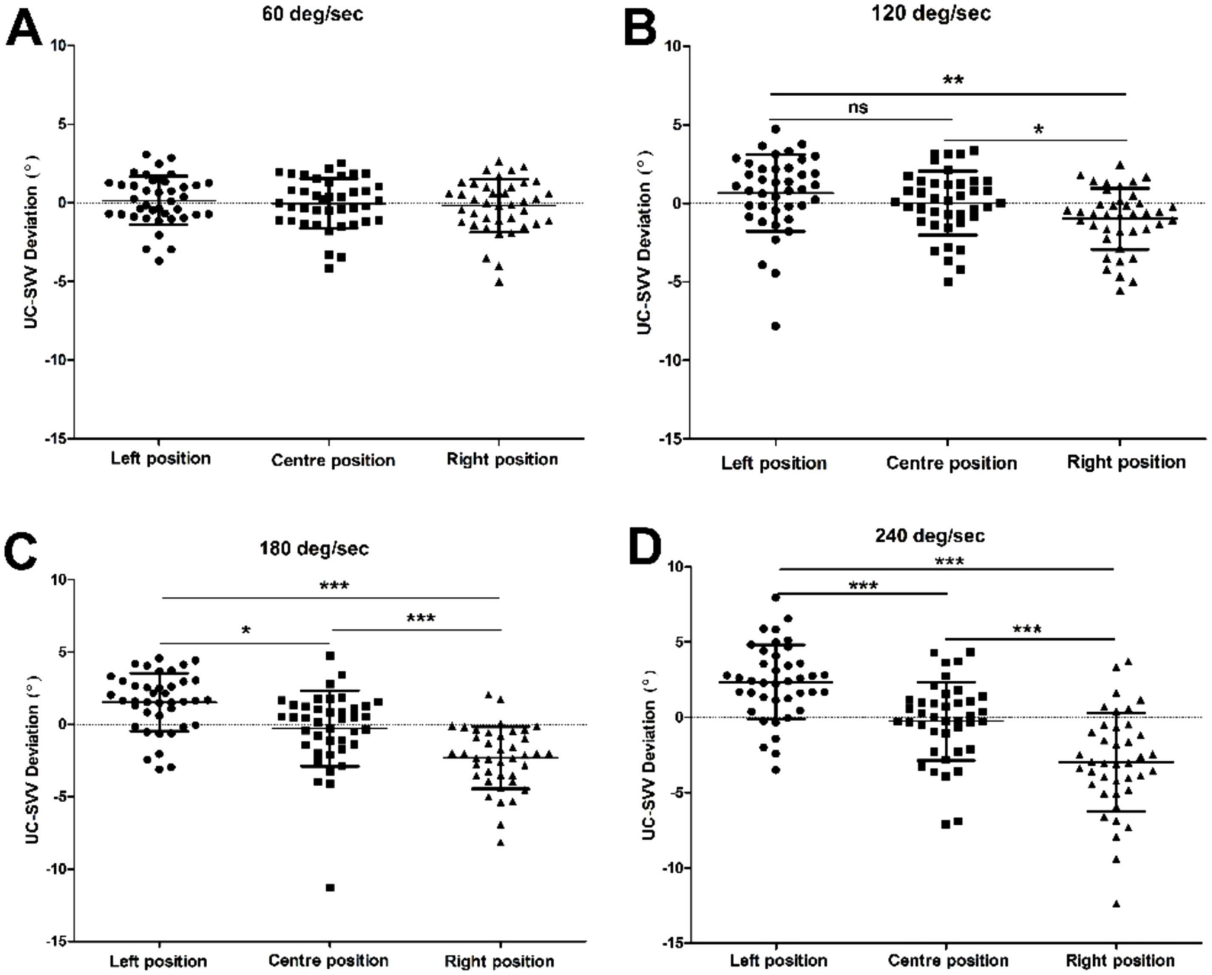
Comparison of the UC-SVV deviation angles at different rotation positions when the rotational stimulus intensity was 60, 120, 180 or 240 deg./s. **(A–D)** Deviation angle of the UC-SVV at different rotation positions when the rotation speed of the rotating chair was 60 deg./s, 120 deg./s, 180 deg./s, and 240 deg./s; **ns**, no significant difference; *p < 0.05; **p < 0.01; ***p < 0.001.

We compared the deviation angle of the UC-SVV line at each rotation position (left/centre/right) at a specific rotation speed (60/120/180/240 deg./s) to analyse the effect of different rotation positions on the deviation angle at a specific rotation stimulus intensity. The results revealed that there was no significant difference in the deviation angle of the UC-SVV line at each rotation position at a rotation speed of 60 deg./s. When the rotation speed was 120 deg./s, the deviation angle of the UC-SVV line was 0.661 ± 2.436 when the rotating chair axis was in the left position and − 0.985 ± 1.947 when the axis was in the right position. There was a significant difference between the two values (*p* < 0.01). However, the deviation angle of the UC-SVV line in the centre position was −0.013 ± 2.033, which was not significantly different from those in the left and right positions. When the rotation speed was 180 deg./s, the deviation angles of the UC-SVV line were 1.523 ± 2.000, −0.281 ± 2.621 and − 2.318 ± 2.141 at the left, centre and right positions, respectively, and there were significant differences among the groups. When the rotation speed was 240 deg./s, the deviation angles of the UC-SVV line were 2.341 ± 2.462, −0.253 ± 2.604 and − 2.976 ± 3.286 at the left, centre and right positions, respectively, and there were significant differences among the groups (Figure 3).

### Influence of different rotation speeds on the deviation angle of the UC-SVV line at specific rotation positions

**Figure 4:**
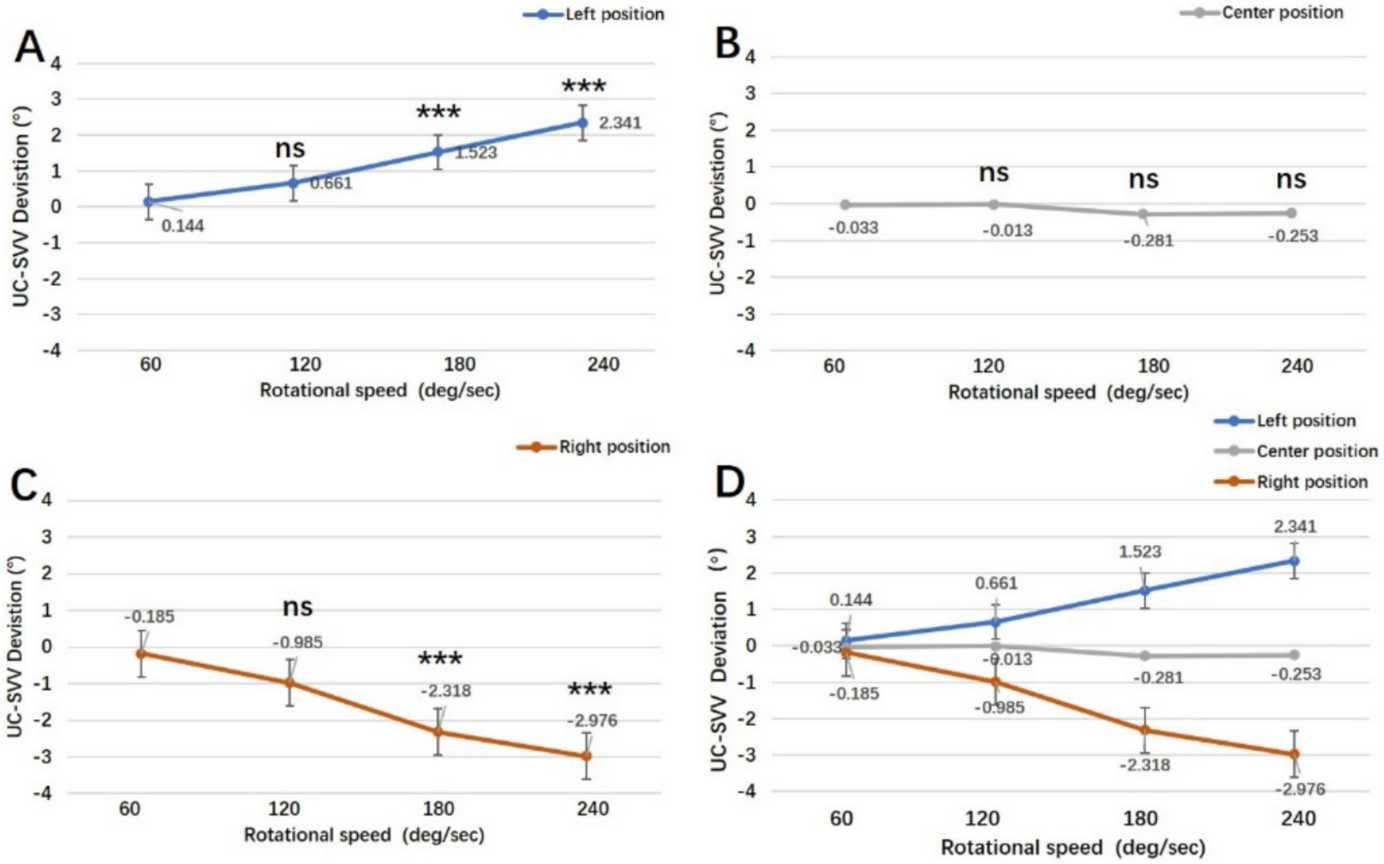
Comparison of the UC-SVV deviation angles at different rotation speeds when the rotation axis was in the left, centre and right positions. **(A–C)** Deviation angle of the UC-SVV at different rotation speeds when the rotation axis was in the left, centre and right positions. **(D)** Comprehensive results at all three positions. ns, no significant difference; ***p < 0.001.

We further compared the deviation angle of the UC-SVV line at different rotation speeds (60/120/180/240 deg./s) at specific rotation positions (right/centre/left position) and analysed the relationship between the UC-SVV deviation and the rotational stimulus intensity at each position. The results revealed that when the rotation chair was translated to the left, the direction of the UC-SVV line deviated to the right, and the deviation angle increased with increasing rotation speed. The deviation angle of the UC-SVV line at 60 deg./s significantly differed from that at 180 and 240 deg./s (*p* < 0.001). When the rotation chair was translated to the right, the UC-SVV line deviated to the left side, and the deviation angle increased with increasing rotation speed. The deviation angle of the UC-SVV line at 60 deg./s significantly differed from that at 180 and 240 deg./s (*p* < 0.001). Previous studies (Chen et al., 2018) have shown that when the rotation speed is 60 deg./s, there is no difference in each rotation position. Therefore, the purpose of this study is to find the effective rotation stimulus, and the research shows that 180/240 deg./s is optimal rotation speed. When the rotation axis was in the centre position, the absolute value of the deviation angle at each speed was less than 0.3, and the difference was not statistically significant (Figure 4).

## Discussion

Spatial position perception and balance regulation of the human body in daily movement mainly rely on the vestibular system. This system includes a semicircular canal and an otolithic subsystem, which can sense angular acceleration (Fetter, 2007) and linear acceleration stimulation (Kingma, 2005), respectively, during human activities. Most of the common vertigo and balance disorders observed in the clinic are caused by vestibular system diseases, and vestibular function assessment is a key step in its diagnosis, treatment and rehabilitation. Traditional vestibular function tests, such as caloric tests, head shaking tests, video head impulse tests and vestibular autorotation tests, all evaluate the semicircular canal system, but the development of otolith system evaluation technology has lagged. At present, the commonly used methods for otolith system function assessment are VEMPs and SVV/SVH (Kingma, 2006; Umibe et al., 2024). VEMPs are short latency surface potentials produced through the activation of saccular and utricular afferents by sound and vibration, which is not consistent with the physiological function of the otolith (Taylor et al., 2020). The SVV/SVH test in peripheral lesions probes static asymmetries in utricular function and represents a perceptual error in perceived gravitational vertical/horizontal direction (Cheng et al., 2022; Zhang et al., 2023). However, the SVV/SVH test involves a vertical/horizontal line adjustment made by patients in a static state, which reflects only bilateral otolith function under static gravitational acceleration. After the static compensation of the vestibular otolith reflex is complete, the SVV/SVH test has difficulty reflecting the pathological status of the bilateral otolith system and is susceptible to interference from various factors, such as vision and ontology.

The UC-SVV test is a novel otolith function detection technology that more closely reflects the physiology of the utricle and can dynamically evaluate the unilateral utriculo-ocular response (UOR; Clarke et al., 2003). In the UC-SVV test, a rotating chair was accelerated to rotate around the axis until reaching a specified speed and continued to rotate at a constant speed for 60 s so that the lymph and ampulla crests in the bilateral lateral semicircular canal moved at a constant speed with the bony semicircular canal to eliminate the effect of this canal. With continued rotation at this speed, the swivel chair was translated to the right by 3.85 cm, with the left utricle of the subject located on the rotation axis, and only the eccentrically positioned utricle (right utricle) was stimulated unilaterally by the resultant centrifugal force (Nowé et al., 2003). Conversely, when the right utricle was located on the axis of rotation, the subject’s left utricle was stimulated. The eccentrically positioned utricle was stimulated by the combined force of gravity and centrifugal force during unilateral centrifugation, namely, gravito-inertial acceleration (GIA), causing the hair cells on that side to deflect and depolarize, resulting in excitatory effects. The subjects mistakenly identified the direction of the GIA as the direction of gravitational acceleration during unilateral centrifugation, produced a new somatic gravity illusion without visual reference, and subsequently created a spatial visual illusion called the oculogravic illusion (Figure 1) to dynamically evaluate the unilateral UOR (Curthoys et al., 1990).

The SVV test is a subjective assessment technique, and parameters such as stimulation intensity and time can affect the accuracy of the test. Gonzalez et al. (2014). explored UC-SVV testing using a chair with a rotation speed of 300 deg./s and a rotation axis at different translation times (5, 10, 15, 20, 25, and 30 s). The results revealed that the shorter the translation time of the rotation axis was, the greater the variability of the test results. Clarke et al. (2003) believed that only a GIA with a rotation speed of 300–400 deg./s could cause significant excitation of the unilateral utricle. Benson et al. (1986) found that the reaction threshold of otoliths is 0.06 m/s^2^, which corresponds to an angular velocity of 53 deg./s. Because the UC-SVV test is time-consuming and often induces various uncomfortable reactions in subjects, it is necessary to carefully consider the test time and stimulation intensity of the UC-SVV test in clinical practice to select the most suitable test conditions to reduce patient discomfort during the test process and improve patient tolerance and the detection accuracy.

In our previous study, a UC-SVV test was performed on healthy subjects with a rotating chair speed of 60 deg./s and a translation time of 30 s (Chen et al., 2018). The results showed that the UC-SVV line deviated when the rotation axis was in the left or right position, but the deviation angle was small, and there was no statistically significant difference compared with that at the centre position, suggesting that the UC-SVV test at this speed lacked practical clinical value. This study further analysed the UC-SVV test with different rotational stimulation intensities of 60, 120, 180, and 240 deg./s. The results showed that the position of the rotational axis (left/centre/right) affected the deviation angle and direction of the UC-SVV line (Figures 2, 3). When the rotational axis was on the left side, the right utricle was stimulated, and the UC-SVV line deviated to the left. Conversely, when the rotational axis was on the right side, the left utricle was stimulated, and the UC-SVV line deviated to the right. With increasing rotation speed, the deviation angle of the UC-SVV line increased (Figure 4), and the angle at 60 deg./s was similar to the results of previous studies (Chen et al., 2018). When the rotation axis was in the centre position, the absolute value of the deviation angle of the UC-SVV line at each rotation speed was less than 0.3, which was not significantly different from the normal value of the traditional static SVV test (Michelson et al., 2018). In addition, Akin et al. (2011) and Gonzalez et al. (2014) both reported that the rotation direction of the chair (clockwise or counterclockwise) did not affect the direction or magnitude of the deviation angle of the UC-SVV line. Therefore, all the subjects were tested with a single clockwise rotation direction in this study.

One of the drawbacks of the use of eccentric rotation is the price of the equipment needed. Helling et al. (2006) indicated that compared SVV in rest and on rotation axis at 240 deg./s indicates then a functional asymmetry of the otolith organs. In their method, rotation stimulated both otolith at the same time, while the eccentric rotation in our study took one side of the otolith as the rotation axis, which enabled dynamic assessment of unilateral otolith function. OCR induced by lateroflexion (Kingma, 1997) is more manoeuverable as it does not require large equipment for the evaluation of otolithic function. However, OCR has higher quality control requirements for subjects receiving homogenised stimulation in clinical practice. In addition to requiring large and expensive rotary chair equipment, this paradigm in our study can test the effectiveness of OCR by performing standard homogeneous stimulation on different subjects and provide evidence for an effective way to explore otolithic function.

### Questions to be studied

As an important balance organ of the human body, otolith function is susceptible to age-related degenerative dysfunction. The threshold of the otolith functional response varies among different age groups. A total of 40 subjects, with a median age of 28 years, were included in this study, and there was no elderly group. In addition, the subjects’ susceptibility to motor disease was not discussed in this study. Therefore, the direction and angle characteristics of UC-SVV lines in the diverse age groups and pathological conditions need to be further studied.

## Conclusion

The UC-SVV test is a new technology for the dynamic evaluation of otolithic function that is based on the rotating chair test. In this study, the reference ranges of the deviation direction and angle of the UC-SVV line were analysed at different rotation speeds (60, 120, 180, 240 deg./s) in healthy subjects. When the peak velocity of rotation was 180 and 240 deg./s, there were significant differences in the deviation angles among the different positions. Therefore, the optimal stimulation intensity of the UC-SVV test is 180 or 240 deg./s. Specifically, the peak speed of rotation could be set at 180 deg./s if the subject is unable to tolerate high-speed rotation. This study provided an objective basis for improving otolith function evaluation technology and its diagnostic significance in vestibular peripheral diseases.

## Data Availability

The raw data supporting the conclusions of this article will be made available by the authors, without undue reservation.
